# New onset, transient and stable motoric cognitive risk syndrome: Clinical characteristics and association with incidence of probable dementia in the NuAge cohort

**DOI:** 10.3389/fnagi.2022.1063702

**Published:** 2023-01-19

**Authors:** Olivier Beauchet, Jacqueline Matskiv, Pierrette Gaudreau, Gilles Allali

**Affiliations:** ^1^Departments of Medicine and Geriatrics, University of Montreal, Montreal, QC, Canada; ^2^Research Centre of the Geriatric University Institute of Montreal, Montreal, QC, Canada; ^3^Department of Medicine, Division of Geriatric Medicine, Sir Mortimer B. Davis Jewish General Hospital and Lady Davis Institute for Medical Research, McGill University, Montreal, QC, Canada; ^4^Research Center of the Centre Hospitalier de l’Université de Montréal, Montreal, QC, Canada; ^5^Leenaards Memory Center, Lausanne University Hospital and University of Lausanne, Lausanne, Switzerland

**Keywords:** older adults, cohort study, dementia, clinical screening, epidemiology

## Abstract

**Background:**

Motoric cognitive risk syndrome (MCR) is a pre-dementia stage. The existence of stable and transient MCR, their related clinical characteristics and their association with incident dementia is a matter of debate.

**Objective:**

This study aims to examine the clinical characteristics and the time course associated with new onset, transient and stable MCR, and their association with incidence of probable dementia in community-dwelling older adults living in the province of Quebec (Canada).

**Design:**

Quebec elderly population-based observational cohort study with 3 years of follow-up.

**Setting:**

Community dwellers.

**Subjects:**

A subset of participants (*n* = 1,113) from the “*Quebec Longitudinal Study on Nutrition and Successful Aging*” (NuAge) cohort.

**Methods:**

Participants with MCR were identified at baseline and after 1 year of follow-up. Socio-demographic characteristics, 30-item Geriatric depression scale (GDS) score, cardiovascular risk factors and diseases were recorded at baseline. Incidence of probable dementia was measured at annual follow-up visits over a 3-year period.

**Results:**

Over the period of the first year of follow-up, the prevalence of MCR was 8.5% with 4.3% having new onset MCR, 2.8% transient MCR and 1.4% stable MCR. A higher 30-item GDS score was reported with new onset and transient MCR, and the highest prevalence of cerebrovascular diseases was shown with stable MCR compared to non-MCR participants (*p* < 0.05). MCR was associated with overall incidence of probable dementia, regardless of its status (Hazard Ratio ≥ 1.86, *p* ≤ 0.034).

**Conclusion:**

Greater prevalence of depressive symptoms and cerebrovascular diseases were reported, respectively, with new onset and transient MCR, and stable MCR. The association of MCR with incidence of probable dementia remains significant, regardless of MCR subtypes.

## Highlights

– Depressive symptoms were more prevalent with new onset and transient Motoric Cognitive Risk syndrome (MCR) compared to non-MCR participants.
– The highest prevalence of cerebrovascular diseases was shown with stable MCR.
– MCR is associated with incidence of probable dementia, regardless of its subtypes (i.e., new onset, transient and stable).

## Introduction

Motoric cognitive risk syndrome (MCR) is a clinical syndrome characterized by slow gait speed and cognitive complaint (Verghese et al., 2013). MCR is an at-risk state for dementia and is proposed to be a transitional state between normal cognition to dementia, such as mild cognitive impairment (MCI; Sekhon et al., 2017). MCR diagnosis, compared to MCI, does not require extensive cognitive testing and neuroimaging, and thus is easy to perform in clinical practice. This enhances its applicability for risk screening of dementia, particularly in the elderly population (Verghese et al., 2013; Sekhon et al., 2017). The usefulness of an MCR diagnosis is two-fold: first, it identifies individuals at risk of dementia and second, it facilitates preventive actions addressing modifiable risk factors among those identified as being at-risk (Verghese, 2021; Mullin et al., 2022). Recent, systematic reviews showed that up to 40% of late-onset dementia may be prevented or at least delayed by addressing modifiable risk factors (Livingston et al., 2020; Curran et al., 2021). MCR has, thus, became a clinical target for more accurate characterization of dementia risk.

The incidence of MCR is high and ranges between 5% and 10% (Verghese et al., 2014a,b; Mullin et al., 2022). Thus, not all individuals with MCR will develop dementia, suggesting that the MCR state may be with time stable, transient or progress to dementia. Since its first description in 2013, no study has examined MCR status may be transient. The possible of transient MCR underscores two main research questions: first, are there clinical characteristics which may explain stable and transient status of MCR, and second, does it influence its predictive value for dementia?

MCR has been associated with cardiovascular risk factors and diseases, as well as brain abnormalities, including microvascular ischemic lesions (Sekhon et al., 2019; Beauchet et al., 2020). In addition, the highest rate of conversion to dementia has been reported for vascular dementia (Verghese et al., 2013). Both characteristics suggest that stable MCR may be associated with cardiovascular risk factors and diseases leading to irreversible brain damage. MCR has also been associated with depressive symptoms (Beauchet et al., 2021; Xu et al., 2022). First, individuals with MCR frequently reported more depressive symptoms compared to their non-MCR counterparts (Beauchet et al., 2021). Second, individuals with depressive symptoms are at risk of MCR (Meiner et al., 2020). As depressive symptoms are reversible, we hypothesized that the reversibility of MCR may be related to these symptoms. The present study thus aims to examine the clinical characteristics and the time course associated with new onset, transient and stable MCR, and the association of these 3 types of MCR with incidence of probable dementia in community-dwelling older adults living in the province of Quebec (Canada).

## Materials and methods

### Design

The database of the “*Quebec Longitudinal Study on Nutrition and Successful Aging”* (NuAge) was used for the present study (Gaudreau et al., 2007). NuAge is a population-based cohort study carried out in the Province of Quebec (Canada) in generally healthy community-dwelling older adults who were evaluated annually over a 3-year period. The data collection of NuAge has been previously reported (Gaudreau et al., 2007). In summary, 1,793 older adults aged between 67 and 84 without cognitive impairment (i.e., Modified Mini-Mental State (3MS) score > 79/100) and without major physical disability (i.e., able to walk 300 m and climb 10 stairs without rest), living independently in the community and willing to commit to up to a 5-year follow-up were recruited between November 2003 and June 2005 (Teng and Chui, 1987; Gaudreau et al., 2007). Among them, 1,753 (97.8%) agreed to the integration of their data and biosamples into the NuAge Database and Biobank for future studies and 1,526 (85.1%) were followed over a 3-year period. Participants with missing values for MCR and dementia status at the end of follow-up were excluded and, thus, 1,113 (62.1%) participants were selected for the present study.

### Assessment

Age, sex, number of reported drugs taken daily were recorded at baseline. Weight (kg) and height (cm) were also measured at baseline. Overweight or obesity was defined as body mass index (BMI) ≥25 kg/m^2^ and underweight was defined as <18.5 kg/m^2^. Currently smoking (yes versus no) was also noted. The Physical Activity Scale for the Elderly (PASE) was used to determine a low physical activity level defined by being below the lowest tertile (i.e., <69.1 for females and <87.7 for males; Washburn et al., 1999). Hypertension was self-reported using the Older Americans Resources and Services Multidimensional Functional Assessment (OARS) questionnaire and defined as the use of anti-hypertensive medications and/or a blood pressure measure >140/90 mmHg (Fillenbaum, 1988). Diabetes, regardless of its type, was recorded if it was self-reported in the OARS questionnaire, or if antidiabetic medications were reported. Data on the presence of cardiovascular diseases associating heart, peripheral and cerebrovascular diseases was collected through the OARS questionnaire. Depressive symptomatology was assessed using the 30-item GDS (Yesavage et al., 1982). Subjective cognitive complaint was recorded and defined as a “yes” response to the question “*Do you feel you have more problems with memory than most*?” from the 30-item GDS and/or as impairment in memory recorded using the memory item of the Functional Autonomy Measurement System (SMAF; Hebert et al., 1988). Finally, gait speed was assessed using a standardized procedure. Participants were asked twice to walk at their usual pace over a 4-m distance. Time was measured in seconds between the second and the fourth meter with a stopwatch and the best time of the two trials was used. Gait speed was calculated in meters per second on the last 3 m. The follow-up period was 3 years. Each year, the full standardized assessment performed at baseline was repeated.

### Definition of motoric cognitive risk syndrome

**Figure 1:**
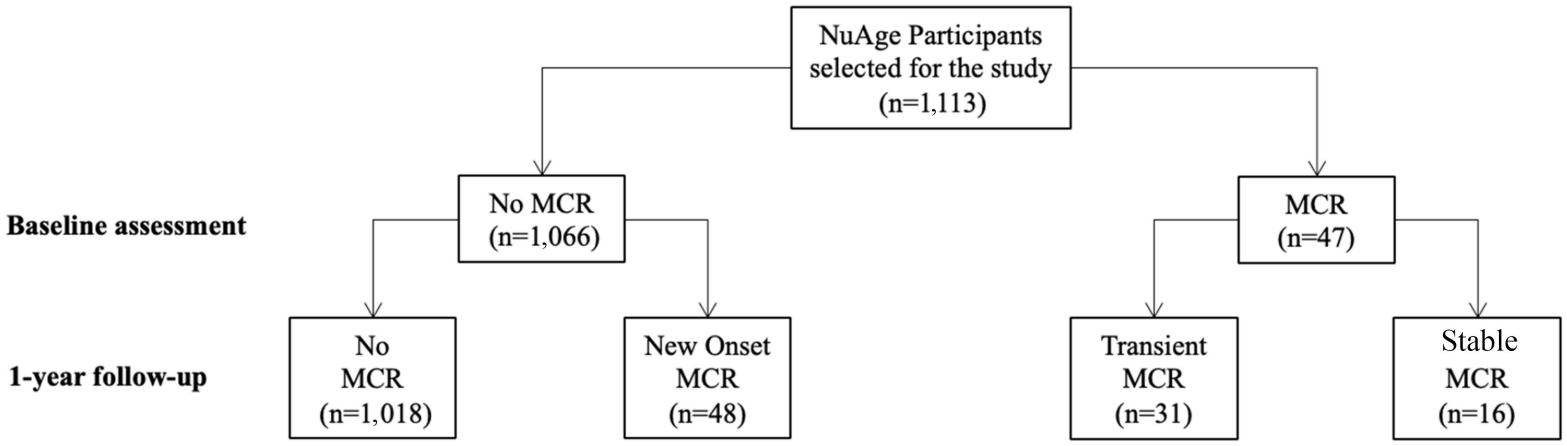
Flow diagram of NuAge participants according to the presence or absence of Motoric cognitive risk syndrome at baseline and 1-year follow-up.

MCR was determined using information collected at baseline and after the first annual follow-up. MCR was defined as a combination of subjective cognitive complaint and slow walking speed in the absence of dementia and gait disability (Verghese et al., 2013). Slow walking speed was defined as a walking speed at least one standard deviation (SD) below the age-appropriate mean values established in the present cohort. Participants were divided into two sex groups and four age groups, as described by Verghese et al. (2013). The slow gait speed cut-offs were: for male <1.09 m/s for age group 67–72, <1.00 m/s for age group 73–77, <0.97 m/s for age group 78–84 and <0.93 m/s for age group ≥85; and for female <1.04 m/s for age group 67–72, <0.97 m/s for age group 73–77, <0.91 m/s for age group 78–84 and <0.81 m/s for age group ≥85. Based on the time course of MCR between baseline assessment and year 1 of follow-up, 3 subtypes of MCR were defined: new onset MCR (no MCR at baseline but MCR after 1 year), transient MCR (MCR at baseline but no MCR after 1 year) and stable MCR (MCR at baseline and after 1 year). Figure 1 shows the distribution of MCR subgroups in selected NuAge participants.

### Definition of probable dementia

Cognitive performance was evaluated at baseline and at each annual subsequent visit using the 3MS score (Teng and Chui, 1987). Incidence of probable dementia was considered present if the 3MS score was ≤79/100, which is a sensitive threshold value to screen dementia, and the simplified Instrumental Activities of Daily Leaving (IADL) score was ≤3/4 (Lawton and Brody, 1969; Teng and Chui, 1987).

### Standard protocol approval and patient consents

The Research Ethics Boards (REB) of the University Institute of Geriatrics of Sherbrooke and the *“Institut universitaire de gériatrie de Montréal”* approved the NuAge protocol. Written informed consent for research was obtained for all NuAge participants. The REB of the *“Centre intégré universitaire de santé et de services sociaux de l’Estrie – Centre hospitalier universitaire de Sherbrooke”(CIUSSS de l’Estrie-CHUS)* approved the NuAge Database and Biobank. The present study was approved by the REB of the Jewish General Hospital (Montreal, Quebec, Canada). The NuAge data set used in this study was obtained from the NuAge Database team on May 07, 2019.

### Statistics

The participants’ baseline characteristics were described using means, standard deviation (SD), and percentages. Participants were assigned to one of the following four groups according to their MCR status: No MCR, new onset MCR, transient MCR and stable MCR. Comparisons between groups were performed using Kruskal-Wallis, Mann–Whitney, Chi-square or Fisher’s exact tests. Cox regressions were also performed to examine the association of MCR subtypes, used as independent variables (separated model for each variable), with incidence of probable dementia. Unadjusted and adjusted models were examined by age, sex, polypharmacy, number of school years, the 30-item GDS and cardiovascular risk factors and diseases. Values of *p* ≤ 0.001 were considered statistically significant for group comparisons (because of multiple comparisons) and those <0.05 were considered significant for Cox regression models. All statistics were performed using SPSS (version 26.0; SPSS, Inc., Chicago, IL).

## Results

Over the period of the first year of follow-up, the prevalence of MCR was 8.5%, of which 4.3% had new onset MCR, 2.8% transient MCR and 1.4% stable MCR. The prevalence of cerebrovascular diseases differed significantly per group (*p* ≤ 0.001; Table 1), with the highest prevalence demonstrated in the stable MCR group (*p* ≤ 0.001; Table 2). The 30-item GDS and 3MS scores were different across MCR subgroups (*p* ≤ 0.001); new onset MCR and transient MCR groups had higher 30-item GDS scores than the non-MCR group (*p* ≤ 0.001). The transient MCR group had a lower 3MS score than its non-MCR counterpart (*p* ≤ 0.001). Both walking speed at baseline and year 1 differed between MCR subgroups, the lowest value reported in the stable MCR group (*p* ≤ 0.001). Similarly, subjective cognitive complaint differed among MCR subgroups, the highest prevalence being reported in stable MCR (*p* ≤ 0.001). Overall incidence of probable dementia also differed among MCR subgroups (*p* ≤ 0.001). It was higher in the transient MCR group compared to the non-MCR group, whereas only trend was reported for the other subgroups (*p* = 0.035 for new onset MCR and *p* = 0.003 for stable MCR).

**Table 1:** Characteristics of participants according to their motoric cognitive risk syndrome status (*n* = 1,113).

	MCR at baseline				Value of *p*^a^
	No (*n* = 1,018)	Yes (*n* = 95)			
		New onset (*n* = 48)	Transient (*n* = 31)	Stable (*n* = 16)	
Age (year), mean ± SD	73.8 ± 4.1	73.8 ± 3.8	75.3 ± 4.3	74.0 ± 3.9	0.293
Female, *n* (%)	526 (51.7)	30 (62.5)	13 (41.9)	9 (56.3)	0.316
Polypharmacy^b^, *n* (%)	445 (43.7)	26 (54.2)	18 (58.1)	11 (68.8)	0.044
Number of school years, mean ± SD	11.9 ± 4.5	11.5 ± 3.5	9.5 ± 4.8	12.8 ± 5.5	0.015
Cardiovascular risk factors, *n* (%)					
Smoking^c^	476 (46.8)	23 (47.9)	15 (48.4)	5 (31.3)	0.660
Overweight/Obesity^d^	733 (72.0)	33 (68.8)	24 (77.4)	10 (62.5)	0.704
Low level of physical activity^e^	328 (32.2)	23 (47.9)	12 (38.7)	7 (43.8)	0.096
Hypertension^f^	469 (46.1)	21 (43.8)	17 (54.8)	8 (50.0)	0.766
Diabetes^g^	97 (9.5)	6 (12.5)	9 (29.0)	2 (12.5)	0.005
≥ 2 risk factors, *n* (%)	617 (60.6)	26 (54.2)	21 (67.7)	8 (50.0)	0.528
Cardiovascular diseases^h^, *n* (%)					
Heart diseases	214 (21.0)	13 (27.1)	5 (16.1)	7 (43.3)	0.100
Limbs vascular diseases	210 (20.6)	17 (35.4)	1 (3.2)	4 (25.0)	0.085
Cerebrovascular diseases	27 (2.7)	3 (6.3)	2 (6.5)	3 (18.8)	**0.001**
≥ 2 diseases, *n* (%)	89 (8.7)	7 (14.6)	0	4 (25.0)	0.072
30-item GDS score (/30), mean ± SD	4.5 ± 3.9	6.2 ± 3.6	7.6 ± 5.0	6.2 ± 4.3	**≤0.001**
3MS score (/100), mean ± SD	94.6 ± 4.0	93.4 ± 4.3	90.7 ± 3.6	91.4 ± 6.2	**≤0.001**
Walking speed, mean ± SD (m/s)					
Baseline	1.18 ± 0.19	1.02 ± 0.18	0.90 ± 0.09	0.82 ± 0.12	**≤0.001**
1-year follow-up	1.17 ± 0.18	0.86 ± 0.08	1.01 ± 0.13	0.86 ± 0.10	**≤0.001**
Cognitive complaint^i^, *n* (%)					
Baseline	189 (18.6)	17 (35.4)	31 (100.0)	16 (100.0)	**≤0.001**
1-year follow-up	256 (25.1)	48 (100.0)	17 (54.8)	16 (100.0)	**≤0.001**
Overall incidence of probable dementia^j^, *n* (%)	19 (1.9)	3 (6.3)	5 (16.1)	2 (12.5)	**≤0.001**

MCR, Motoric Cognitive Risk; GDS, geriatric depression scale; 3MS, Modified Mini-Mental State.

a Kruskal-Wallis, Chi-square test or Fisher’s exact tests were used for group comparisons, as appropriate.

b Number of therapeutic drugs taken daily ≥ 5.

c Currently smoking, coded as binary answer (yes versus no).

d Considered if BMI was ≥ 25 kg/m^2^.

e Score below the lowest tertile (i.e., <69.1 for female and <87.7 for male).

f Self-reported, noted positive on the Older Americans Resources and Services Multidimensional Functional Assessment (OARS) questionnaire, anti-hypertensive medications were being used, or with a blood pressure measurement > 140/90 mmHg.

g Self-reported, noted positive on the OARS questionnaire, antidiabetic medication was reported, or with a fasting glycemia > 6.9 mmol/L.

h Based on the OARS questionnaire information, 3 dichotomous (i.e., yes versus no) categories of diseases were created to indicate the presence of cardiovascular diseases (associating heart, limb and cerebrovascular diseases).

i Answer to the 30-item Geriatric Depression Scale “*Do you feel you have more problems with memory than most others?*” yes and/or as impairment in memory recorded using the memory item of Functional Autonomy Measurement System (SMAF).

j Modified Mini-Mental State score ≤ 79/100 and the simplified Instrumental Activities of Daily Leaving score ≤ 3/4; significant *p*-values (i.e., <0.0025 due to the multiple comparisons *n* = 20) indicated in bold.

**Table 2:** Value of *p* comparisons between groups of participants defined according to their motoric cognitive risk syndrome status at baseline (*n* = 1,113).

	At baseline assessment			Transient MCR versus Stable MCR
	No MCR versus				New onset MCR versus	
	New onset MCR	Transient MCR	Stable MCR		Transient MCR	Stable MCR
Age (year)	0.908	0.557	0.780	0.109	0.864	0.311
Female	0.142	0.285	0.716	0.073	0.657	0.351
Polypharmacy^a^	0.154	0.113	0.045	0.773	0.306	0.475
Number of school years	0.859	0.002	0.470	0.009	0.400	0.075
Cardiovascular risk factors						
Smoking^b^	0.875	0.858	0.217	0.967	0.244	0.260
Overweight/Obesity^c^	0.624	0.508	0.402	0.401	0.645	0.279
Low level of physical activity^d^	0.024	0.447	0.328	0.421	0.772	0.739
Hypertension^e^	0.753	0.335	0.754	0.335	0.664	0.753
Diabetes^f^	0.496	**≤0.001**	0.689	0.067	1.000	0.205
≥ 2 risk factors	0.373	0.423	0.389	0.230	0.772	0.236
Cardiovascular diseases^g^						
Heart diseases	0.316	0.509	0.028	0.257	0.213	0.040
Limbs vascular diseases	0.014	0.541	0.668	0.062	0.442	0.464
Cerebrovascular diseases	0.141	0.845	**≤0.001**	0.549	0.137	0.071
≥ 2 diseases	0.167	0.655	0.024	0.267	0.339	0.071
30-item GDS score (/30)	**≤0.001**	**≤0.001**	0.058	0.321	0.797	0.344
3MS score (/100)	0.050	**≤0.001**	0.035	0.002	0.307	0.265
Walking speed (m/s)						
Baseline	**≤0.001**	**≤0.001**	**≤0.001**	0.003	**≤0.001**	0.012
1-year follow-up	**≤0.001**	**≤0.001**	**≤0.001**	**≤0.001**	0.975	**≤0.001**
Cognitive complaint^h^						
Baseline	0.004	**≤0.001**	**≤0.001**	**≤0.001**	**≤0.001**	–
1-year follow-up	**≤0.001**	**≤0.001**	**≤0.001**	**≤0.001**	–	**≤0.001**
Overall incidence of probable dementia^i^	0.035	**≤0.001**	0.003	0.149	0.434	0.768

MCR, Motoric Cognitive Risk; GDS, geriatric depression scale; 3MS, Modified Mini-Mental State; Comparisons based on Mann–Whitney tests or Chi-squared test or Fisher’s exact test, as appropriate.

a Number of therapeutic drugs taken daily ≥ 5.

b Currently smoking, coded as binary answer (yes versus no).

c Considered if BMI was ≥25 kg/m^2^.

d Score below the lowest tertile (i.e., <69.1 for female and <87.7 for male).

e Self-reported, noted positive on the Older Americans Resources and Services Multidimensional Functional Assessment (OARS) questionnaire, anti-hypertensive medications were being used, or with a blood pressure measurement > 140/90 mmHg.

f Self-reported, noted positive on the OARS questionnaire, antidiabetic medication was reported, or with a fasting glycemia > 6.9 mmol/L.

g Based on the OARS questionnaire information, 3 dichotomous (i.e., yes versus no) categories of diseases were created to indicate the presence of cardiovascular diseases (associating heart, limb and cerebrovascular diseases).

h Answer to the 30-item Geriatric Depression Scale “*Do you feel you have more problems with memory than most others?*” yes and/or as impairment in memory recorded using the memory item of Functional Autonomy Measurement System (SMAF).

i Modified Mini-Mental State score ≤ 79/100 and the simplified Instrumental Activities of Daily Leaving score ≤ 3/4; significant *p*-values (i.e., ≤0.001 due to the multiple comparisons *n* = 120) indicated in bold.

Unadjusted Cox regressions revealed that transient and stable MCR (Hazard ratio (HR) ≥ 1.88 with *p* ≤ 0.011; Table 3) were associated with incidence of probable dementia. Adjusted Cox regressions showed that all MCR subtypes were associated with incidence of probable dementia (HR ≥ 1.86 with *p* ≤ 0.034).

**Table 3:** Cox regressions showing the association of Motoric Cognitive Risk syndrome status (i.e., new onset, transient and stable used as independent, separated model) and overall incidence of probable dementia (used as dependent variable; *n* = 1,113).

	Dementia					
	Unadjusted model			Adjusted model		
	HR	[95% CI]	Value of *p*	HR	[95% CI]	Value of *p*
New onset MCR	3.38	[1.00; 11.41]	0.050	3.78	[1.11; 12.88]	**0.034**
Transient MCR	2.97	[1.81; 4.86]	**≤0.001**	2.17	[1.30; 3.63]	**0.003**
Stable MCR	1.88	[1.16; 3.05]	**0.011**	1.86	[1.14; 3.05]	**0.013**

MCR, motoric cognitive risk syndrome; HR, Hazard ratio; ^*^Adjusted by age, sex, polypharmacy, number of school years, the 30-item GDS and cardiovascular risk factors and diseases; CI, Confidence interval; value of *p* significant (i.e., <0.05) indicated in bold.

## Discussion

Our findings show that MCR may be transient and that specific characteristics are related to this reversibility. Depressive symptoms were more prevalent in participants with new onset and transient MCR, whereas cerebrovascular diseases were more prevalent in participants with stable MCR. In addition, the MCR subtypes did not influence the association with incidence of probable dementia, which was significant in all subtypes.

The reversibility from MCR to a normal condition has not been previously examined. We reported that there are individuals with transient MCR after only 1 year of follow-up. MCR is a pre-dementia stage like MCI. MCI reversibility has previously been reported in community-based cohorts (Malek-Ahmadi, 2016; Gao et al., 2018). Both MCR and MCI are transitional stages between normal cognition and dementia, and their definition is based on clinical characteristics which may change over the time. The impact of interindividual variability on clinical characteristics may be important when dichotomous outcomes are used (Haidich, 2010). Both improvement in medical conditions and the practice effect, which refers to improvement in test performance because of prior exposure to testing, are individual-based factors that may lead to the reversibility of MCR, especially if the disease related to MCR is transitional like depression compared to irreversible conditions, such as cerebrovascular brain lesions (Haidich, 2010; Tractenberg and Pietrzak, 2011).

We reported in the present study that both reversibility of MCR to a normal condition and participants with new onset MCR had a higher 30-item GDS score compared to their counterparts. The relationship of MCR and depressive symptoms has previously been reported (Sekhon et al., 2019; Beauchet et al., 2021; Xu et al., 2022). For instance, we observed that there was a higher prevalence of depressive symptomatology in adults with MCR compared to those without MCR in the Canadian population (Sekhon et al., 2019). Furthermore, it has been demonstrated that depressive symptoms are associated with incident MCR (Xu et al., 2022). Our finding underscores a new component of the relationship between depressive symptoms and MCR, which is the reversibility of MCR. A possible explanation of this characteristic may be related to the reversibility of depressive symptoms. Both individuals with MCR and depressive symptoms report subjective cognitive complaint (Sekhon et al., 2019; Beauchet et al., 2021; Xu et al., 2022). In addition, individuals with depressive symptoms may have a slow walking speed compared to their healthy counterparts (Sekhon et al., 2019; Xu et al., 2022). Thus, we suggest that the cause of MCR in individuals with transient and new onset MCR may be attributed to the depressive symptoms, and that the fluctuation of depressive symptomatology with time may explain the fluctuation of MCR diagnosis.

In contrast to transient and new onset MCR, individuals with stable MCR had a higher prevalence of cerebrovascular diseases when compared to non-MCR individuals. First, this finding is consistent with a previous meta-analysis which showed that MCR was significantly associated with cardiovascular diseases and risk factors (Beauchet et al., 2018). Second, the stability of diagnosis of MCR with time suggests that MCR may be related to irreversible brain lesions. Unfortunately, it is not possible to confirm this hypothesis with the NuAge study because we do not have a brain imaging. MCR has been associated with both cortical atrophy and microvascular ischemic brain abnormalities (Beauchet et al., 2018; Blumen et al., 2021). In addition, it has been shown that MCR is a greater predictor of vascular dementia compared to Alzheimer’s disease (Verghese et al., 2013; Sekhon et al., 2017; Verghese, 2021; Mullin et al., 2022). For instance, the risk of developing dementia reported in the first publication involved a hazard ratio [HR] of 3.3 (95% Confidence interval (CI): 1.55–6.90), which increased to 12.8 (95% CI: 4.98–32.97) for vascular dementia (Verghese et al., 2013). When combined, these previous results and our present findings suggest that the mechanism of stable MCR may be related to cerebrovascular lesions.

Finally, we found that regardless of its subtype (i.e., new onset, transient or stable), MCR is significantly associated with incidence of probable dementia. This result confirms the strong relationship between MCR and dementia reported in a recent meta-analysis (Mullin et al., 2022). The novelty of our results lies in the fact that the reversibility of MCR did not influence the occurrence of probable dementia. We suggested that the reversibility of MCR is related to depressive symptoms. A systematic review and meta-analysis of community-based cohort studies has shown that late-life depressive symptomatology is associated with an increased risk for incident dementia, regardless of its type (i.e., Alzheimer’s disease (AD) and non-AD; Diniz et al., 2013).

The present study has some limitations. First, the NuAge population was composed of relatively healthy older adults, which could affect the generalization of our results. Second, although we were able to control for many characteristics likely to modify the association, residual confounding might still be present. As confounding factors can impact both the magnitude and direction of the association, it is difficult to speculate on the impact of residual confounding factors on the associations found in the study. Third, we selected 62.1% of participants from the initial set of NuAge cohort due to selection criteria and accessible data for this study, that may be considered as a selection bias. Indeed, the sample selection of this study may not accurately reflect the older population. Fourth, the diagnosis of dementia may be underestimated, explaining its low incidence. Indeed, this diagnosis is usually based on an interdisciplinary meeting and more exhaustive information. In our case, we used the dementia threshold of the 3MS combined with an abnormal simplified IADLs score. Fifth, there is no brain imaging or other biomarker variables that can be used as a reference indicator for dementia risk or neurodegeneration/pathology status (e.g., lesions or vascular problems).

## Conclusion

Our study showed that depressive symptoms were more prevalent with new onset and transient MCR, whereas cerebrovascular diseases were more prevalent with stable MCR in Quebec community-dwelling older adults. The MCR subtype status did not influence its association with incidence of probable dementia.

## Data availability statement

The data analyzed in this study is subject to the following licenses/restrictions: Access to this research bank can be requested by completing an access request on the NuAge Database and Biobank website (https://nuage.recherche.usherbrooke.ca/en/). Requests to access these datasets should be directed to https://nuage.recherche.usherbrooke.ca/en/.

## Ethics statement

The studies involving human participants were reviewed and approved by The Research Ethics Boards (REB) of the University Institute of Geriatrics of Sherbrooke and the “Institut universitaire de gériatrie de Montréal.” The patients/participants provided their written informed consent to participate in this study.

## Author contributions

OB and GA: conceived and designed the experiments, analyzed and interpreted the data. PG: performed the experiments. PG and OB: contributed reagents, materials, and analysis tools or data. OB, JM, and GA: writing of the manuscript. PG: revision of manuscript. All authors contributed to the article and approved the submitted version.

## Funding

The NuAge Study was funded by the Canadian Institutes of Health Research (CIHR; MOP-62842). The NuAge Database and Biobank are supported by the Fonds de recherche du Québec (FRQ; 2020-VICO-279753), the Quebec Network for Research on Aging, a thematic network funded by the FRQ-Santé, and by the Merck-Frosst Chair funded by La Fondation de l’Université de Sherbrooke. Beauchet and Allali were supported by the National Institute of Health/National Institute on Aging grants PO1 AG03949 and R01AG057548-01A1. The sponsors had no role in the design, execution, analysis and interpretation of data, or writing of the study.

## Conflict of interest

The authors declare that the research was conducted in the absence of any commercial or financial relationships that could be construed as a potential conflict of interest.

## Publisher’s note

All claims expressed in this article are solely those of the authors and do not necessarily represent those of their affiliated organizations, or those of the publisher, the editors and the reviewers. Any product that may be evaluated in this article, or claim that may be made by its manufacturer, is not guaranteed or endorsed by the publisher.
